# Performance of a Deep Learning Model vs Human Reviewers in Grading Endoscopic Disease Severity of Patients With Ulcerative Colitis

**DOI:** 10.1001/jamanetworkopen.2019.3963

**Published:** 2019-05-17

**Authors:** Ryan W. Stidham, Wenshuo Liu, Shrinivas Bishu, Michael D. Rice, Peter D. R. Higgins, Ji Zhu, Brahmajee K. Nallamothu, Akbar K. Waljee

**Affiliations:** 1 Michigan Integrated Center for Health Analytics and Medical Prediction (MiCHAMP), University of Michigan, Ann Arbor; 2 Division of Gastroenterology and Hepatology, Department of Internal Medicine, University of Michigan, Ann Arbor; 3 Department of Statistics, University of Michigan, Ann Arbor; 4 Division of Cardiology, Department of Internal Medicine, University of Michigan, Ann Arbor; 5 Veterans Affairs Center for Clinical Management Research, Ann Arbor, Michigan; 6 Department of Internal Medicine, Veteran Affairs Ann Arbor Health Care System, Ann Arbor, Michigan

## Abstract

**Importance:**

Assessing endoscopic disease severity in ulcerative colitis (UC) is a key element in determining therapeutic response, but its use in clinical practice is limited by the requirement for experienced human reviewers.

**Objective:**

To determine whether deep learning models can grade the endoscopic severity of UC as well as experienced human reviewers.

**Design, Setting, and Participants:**

In this diagnostic study, retrospective grading of endoscopic images using the 4-level Mayo subscore was performed by 2 independent reviewers with score discrepancies adjudicated by a third reviewer. Using 16 514 images from 3082 patients with UC who underwent colonoscopy at a single tertiary care referral center in the United States between January 1, 2007, and December 31, 2017, a 159-layer convolutional neural network (CNN) was constructed as a deep learning model to train and categorize images into 2 clinically relevant groups: remission (Mayo subscore 0 or 1) and moderate to severe disease (Mayo subscore, 2 or 3). Ninety percent of the cohort was used to build the model and 10% was used to test it; the process was repeated 10 times. A set of 30 full-motion colonoscopy videos, unseen by the model, was then used for external validation to mimic real-world application.

**Main Outcomes and Measures:**

Model performance was assessed using area under the receiver operating curve (AUROC), sensitivity and specificity, positive predictive value (PPV), and negative predictive value (NPV). Kappa statistics (κ) were used to measure agreement of the CNN relative to adjudicated human reference cores.

**Results:**

The authors included 16 514 images from 3082 unique patients (median [IQR] age, 41.3 [26.1-61.8] years, 1678 [54.4%] female), with 3980 images (24.1%) classified as moderate-to-severe disease by the adjudicated reference score. The CNN was excellent for distinguishing endoscopic remission from moderate-to-severe disease with an AUROC of 0.966 (95% CI, 0.967-0.972); a PPV of 0.87 (95% CI, 0.85-0.88) with a sensitivity of 83.0% (95% CI, 80.8%-85.4%) and specificty of 96.0% (95% CI, 95.1%-97.1%); and NPV of 0.94 (95% CI, 0.93-0.95). Weighted κ agreement between the CNN and the adjudicated reference score was also good for identifying exact Mayo subscores (κ = 0.84; 95% CI, 0.83-0.86) and was similar to the agreement between experienced reviewers (κ = 0.86; 95% CI, 0.85-0.87). Applying the CNN to entire colonoscopy videos had similar accuracy for identifying moderate to severe disease (AUROC, 0.97; 95% CI, 0.963-0.969).

**Conclusions and Relevance:**

This study found that deep learning model performance was similar to experienced human reviewers in grading endoscopic severity of UC. Given its scalability, this approach could improve the use of colonoscopy for UC in both research and routine practice.

## Key Points

**Question:**

What is the agreement of automatically determined endoscopic severity of ulcerative colitis using deep learning models compared with expert human reviewers?

**Findings:**

In this diagnostic study including colonoscopy data from 3082 adults, performance of a deep learning model for distinguishing moderate to severe disease from remission compared with multiple expert reviewers was excellent, with an area under the receiver operating curve of 0.97 using still images and full-motion video.

**Meaning:**

Deep learning offers a practical and scalable method to provide objective and reproducible assessments of endoscopic disease severity for patients with ulcerative colitis.

## Teaser

This diagnostic study compares an automated deep learning model with experienced human reviewers in assessment of endoscopic diseases of adults with severity of ulcerative colitis.

## Introduction

Grading endoscopic severity of disease is critical for evaluating response to therapy in patients with ulcerative colitis (UC). Endoscopic severity correlates with patient symptoms and predicts long-term clinical outcomes and the need for additional treatments.^1,2,3^ Although several scoring systems for tracking patients are available, the full Mayo score is the most widely used because it incorporates features collected during colonoscopy with additional patient-reported symptoms.^4,5^ As a result of these features, the full Mayo score is used to determine candidacy for and efficacy of new therapeutics in both European and North American clinical trials.^6,7^ Yet use of the Mayo subscore, the endoscopic component alone, in routine practice is limited by insufficient availability of experienced human reviewers adequately trained to apply it in a standardized manner.^8^

**Figure 1.**
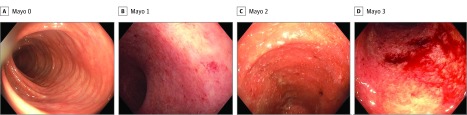
Mayo Endoscopic Subscore Descriptors and Representative Images Endoscopic image features including degree of erythema, visible vascular pattern, friability, ulceration, and spontaneous bleeding are used to categorize Mayo subscore. A, Mayo 0: no friability or granularity; intact vascular pattern. B, Mayo 1: erythema; Decreased vascular pattern; mild friability. C, Mayo 2: marked erythema; absent vascular pattern; friability; erosions. D, Mayo 3: marked erythema; absent vascular pattern; friability; granularity; spontaneous bleeding; ulcerations.

Although its output is quantitative, the endoscopic scoring relies on subjective interpretation by individual operators of images obtained during colonoscopy (Figure 1). Local site scoring, despite being performed by inflammatory bowel disease (IBD) specialists, has been shown to have substantial interobserver and intraobserver variability in the grading of endoscopic severity.^9^ In addition, local site investigators tend to systematically overscore baseline endoscopic severity in IBD compared with remote investigators.^10^ Endoscopic score reproducibility, reliability, and objectivity have been improved with the use of central reading by experienced and trained reviewers uninvolved in direct patient care.^11^ Although essential and feasible in the research setting, central review of colonoscopy is impractical for use in the clinical domain for standardized decision making owing to insufficient access to the necessary volume of experienced readers and high financial costs.

Advances in artificial intelligence methods are increasingly being used across fields of medicine to automate image analysis. Specifically, recent examples include deep learning algorithms for diagnosing melanoma, diabetic retinopathy, polyps, and other conditions from still images.^12,13,14,15,16^ Application of similar methods to images acquired from videos, such as those obtained during colonoscopy, is early in development. However, still images are potentially useful in conditions such as UC because they could provide an accurate, broadly accessible, and low-cost tool for research and clinical applications. We investigated the feasibility of deep learning algorithms to grade endoscopic severity of UC and applied it to full-motion video recordings of colonoscopies.

## Methods

### Study Cohort and Image Selection and Labeling

Patients with UC who underwent endoscopy (colonoscopy or flexible sigmoidoscopy) between January 1, 2007, and December 31, 2017, were identified from the electronic health records of the University of Michigan Health System, a large tertiary care center in the United States. A clinical diagnosis of UC was determined using a previously validated definition that requires 2 *International Classification of Diseases, Ninth Edition* (*ICD-9*) or *ICD-10* (after October 1, 2015) diagnosis codes for UC on 2 separate encounters and at least 1 record of use of medication for UC.^17^ We excluded any patients with *ICD-9* or *ICD-10* diagnosis codes for Crohn disease or a *Current Procedural Terminology* (*CPT*) code before the date of colonoscopy indicating colectomy, ileoanal pouch anastomosis, colostomy, ileostomy, or other bowel resection. Specific inclusion and exclusion codes, as well as a list of UC medications, are provided in the eMethods in the Supplement. All patients meeting these criteria were included to generate a cohort that approximated the distribution of disease severity seen in the general population of patients with UC. This study protocol was approved by the University of Michigan Institutional Review Board. Consent was waived in the still-image data set, because this used retrospective data from over 10 years. However, constructively enrolled patients for the video set provided consent at the time of video recording. This study followed the Standards for Reporting of Diagnostic Accuracy (STARD) reporting guideline.

Endoscopic still images from the colonoscopy reports of patients with UC who met selection criteria were retrieved from the University of Michigan Endoscopic Database, a repository for digital endoscopic images. The endoscopic still images were collected and then provided to 2 independent physician reviewers (R.W.S., S.B.) with experience using the Mayo subscore in therapeutic clinical trials. Human reviewers were blinded to all participant clinical details and identifying information. Images were graded for Mayo endoscopic severity, with scoring disagreement between reviewers identified and a final reference score adjudicated by a third independent reviewer (M.D.R.). The adjudicated scores from this endoscopic still-image data set provided the ground truth for endoscopic scoring model development.

To evaluate the performance of automated scoring models, a second set of still endoscopic images that were not used in model development was obtained from full-motion colonoscopy videos. Colonoscopy videos were recorded in consecutive patients presenting for UC evaluation. Videos were partitioned into still images at 1 frame per second with each image scored by human reviewers and the automated scoring model. Eligible UC patients had videos recorded using a CF-HQ190 or PCF-H190 colonoscope with CLV-190 image processors (Olympus Corp Inc) at 1920 × 1080 resolution, 10-bit color depth, and 60 frames per second.

### Model Generation

Images from the endoscopic still-image data set were split at the patient level with random allocation into a training set (80% used for model building and 10% used to tune model hyperparameters) and a testing set (10% unseen in model development used to evaluate final model performance). Source images were downscaled to 320 × 256 resolution and underwent random transformations of rotation, zoom, sheer, and vertical and horizontal orientation to improve the variability of the data set and prevent overfitting. A convolutional neural network (CNN) was used, implementing an image classification architecture based on Inception V3, a 159-layer CNN.^18^ The CNN model was initialized using weights pretrained on ImageNet^19^ followed by end-to-end training using adaptive moment estimation.^20^ Model output generated the probabilities of each Mayo subscore for each image based on binary classifications with ordinal characteristics of the Mayo score assumed. The ordinal binary classifiers were combined to predict the highest probability Mayo subscore for each image using the methods developed by Cardoso and Pinto da Casa^21^ for classifying ordinal data. For our primary classification task, we predicted normal to mild (Mayo 0 or 1 endoscopic score) vs moderate to severe (Mayo 2 or 3 endoscopic score). We chose this classification because it has been used by the US Food and Drug Administration and the European Medicines Agency for tracking disease severity and is a recommended decision point in many therapeutic clinical trials.^6,7^ Convolutional neural network model building was performed using Tensorflow and Keras packages in Python 3.5 (Python Software Foundation). A 10-fold cross-validation was performed with 95% CIs calculated based on a *t* distribution.

### Statistical Analysis

Agreement on exact Mayo scores for still images between CNN prediction and adjudicated human reviewer scores used the Cohen κ coefficient; this was also used to assess interrater agreement between human reviewers. Differences in proportion of agreement by Mayo score level were assessed using the χ^2^ test with 2-sided *P* < .05 used as the level of statistical significance. Areas under the receiver operating curve (AUROCs) were generated to measure the accuracy in discrimination of the CNN relative to the adjudicated human reference score. We also calculated additional test performance characteristics of sensitivity, specificity, positive predictive value, and negative predictive value with 95% CIs reported. Test performance measures underwent 10-fold cross-validation using test images unseen in model development.

As an exploratory pilot, we also attempted to determine the overall summary Mayo subscores for complete colonoscopy videos. The summary Mayo score is assigned based on the greatest severity detected by the performing physician, independent of the distribution or length of disease. Human reviewers provided summary Mayo scores after viewing complete colonoscopy videos. Using the developed CNN model to classify the Mayo score for each individual frame of colonoscopy video, a predicted overall summary Mayo score of 0, 1, 2, or 3 was generated. Predicted summary scores relied on the proportion of video frames Mayo scores. The still-frame score proportions for human summary Mayo scores were used to inform the frame proportions used for automated summary Mayo scoring; the highest score meeting threshold proportion criteria was selected. The still-frame score proportion thresholds used for automated summary Mayo scores were as follows: Mayo 3 if more than 10% of video still frames were scored Mayo 3, Mayo 2 if more than 20% of video still frames were scored Mayo 2, Mayo 1 if more than 30% of video still frames were scored Mayo 1, and Mayo 0 if none of the criteria were met.

## Results

### Study Cohort

**Figure 2.**
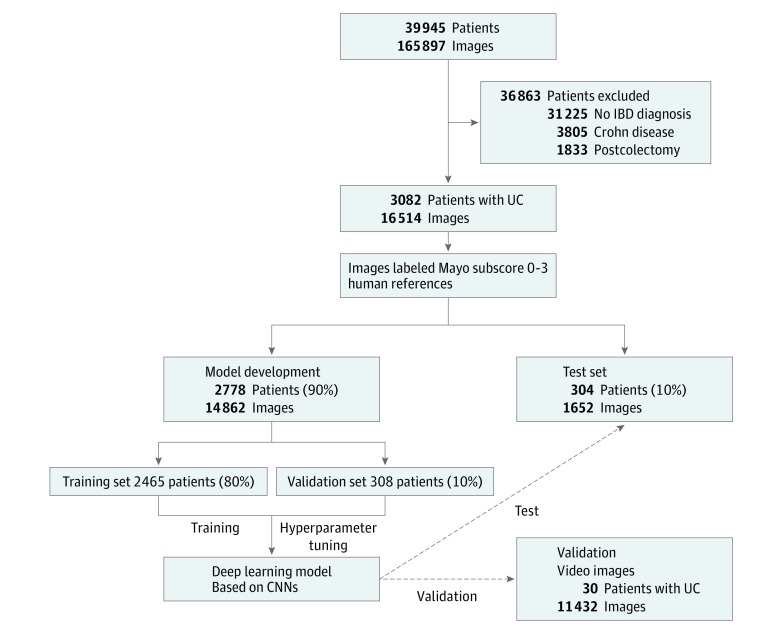
Schematic of Deep Learning Model for Predicting Mayo Endoscopic Score Archived still images from earlier colonoscopies of patients with ulcerative colitis (UC) who met selection criteria were independently scored (labeled) for Mayo endoscopic score by 2 independent gastroenterologists specializing in inflammatory bowel disease (BD). Scored still images were randomly split (by patients) into a model development set and a test set. Resulting deep learning models were applied to the test set and a separate validation set of still images collected from 30 colonoscopy videos not used in model building. CNN indicates convolutional neural networks.

Patient selection criteria resulted in 3082 unique patients with UC (Figure 2). Among these patients, the median (IQR) age was 41.3 (26.1-61.8) years and 1678 (54.4%) were female (eTable 1 in the Supplement). A total of 16 514 unique images were used in the analysis, with a distribution of Mayo subscores of 0 of 8951 (54.2%); 1, 3584 (21.7%); 2, 2278 (13.8%); and 3, 1701 (10.3%). The testing set was a random 10% sample, split by patients, not images, and consisted of 1652 images from 304 unique patients. Human reviewers agreed on exact Mayo subscore in 11907 images (72.1%), leaving 4607 images (27.9%) for score adjudication by a third reviewer. Among reviewer disagreements, 91.5% were by 1 score level. Human reviewers were significantly more likely disagree on intermediate Mayo scores of 1 and 2 compared with extreme Mayo scores of 0 and 3 (38.7% vs 21.8%; *P* < .001).

### Model Performance Compared With Human Endoscopic Scoring

**Figure 3.**
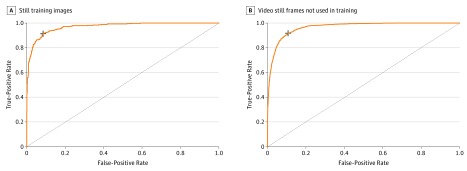
Convolutional Neural Networks (CNNs) for Automated Identification of Endoscopic Remission of Ulcerative Colitis A, A CNN was trained on reference colonoscopy images scored by 2 independent reviewers, with adjudication of disagreements by a third reviewer. CNN discrimination between endoscopic remission (Mayo 0 or 1) from moderate to severe activity (Mayo 2 or 3) had an area under the receiver operating curve (AUROC) of 0.97. B, The CNN had similar performance differentiating remission from moderate to severe disease in a separate set of images from colonoscopy videos not used in model building, with an AUROC of 0.97. Dashed lines represent a nondiscriminatory AUROC. Plus sign indicates optimal sensitivity and specificity.

Based on US Food and Drug Administration and European Medicines Agency guidance for defining endoscopic remission (Mayo subscore of 0 or 1) compared with moderate to severe disease (Mayo subscore of 2 or 3), we found model prediction was excellent, with an AUROC of 0.970 (95% CI, 0.967-0.972) (Figure 3A). Model performance characteristics for separating Mayo subscore of 0 or 1 vs 2 or 3 were very good, with a sensitivity of 83.0% (95% CI, 80.8%-85.4%) and specificity of 96.0% (95% CI, 95.1%-97.1%); positive predictive value of 0.86 (95% CI, 0.85-0.88); and negative predictive value of 0.94 (95% CI, 0.93-0.95).

Comparing agreement on exact endoscopic Mayo subscore, the agreement between the CNN and the adjudicated human reference score was similar to the agreement observed between individual reviewers (κ = 0.84 vs κ = 0.86, respectively). Mayo scores of 0 predicted by the CNN exactly matched adjudicated scores in 89.6% of cases; Mayo 1 in 54.5% of cases; Mayo 2 in 69.9% of cases; and Mayo 3 in 74.3% of cases (Table 1). Comparatively, agreement between human reviewers for Mayo scores of 0 occurred in 77.1% of cases; Mayo 1 in 54.7% of cases; Mayo 2 in 69.3% of cases; and Mayo 3 scores in 85.7% of cases (Table 2).

**Table 1.** Agreement Between Adjudicated Human Reviewer Scores and Automated Mayo Subscore Within an Endoscopic Still-Image Testing Data Set

Human Mayo Score^a^	Predicted Mayo Score, %				Human Total No. of Images Reviewed
	0	1	2	3	
0	89.6	8.2	2.0	0.2	922
1	30.1	54.5	12.7	1.6	299
2	5.0	16.4	69.9	8.6	256
3	0.5	0.6	24.6	74.3	175
Predicted total, No.	942	282	270	158	1652

^a^ Increasing Mayo endoscopic scores denote increasing mucosal inflammation in the colon, where a score of 0 indicates normal-appearing colonic mucosa and 3 indicates severe inflammatory changes.

**Table 2.** Agreement Between Individual Human Reviewers Within an Endoscopic Still-Image Data Set

Reviewer A Mayo Score^a^	Reviewer B Mayo Score, %				Reviewer B, No. of Images Reviewed
	0	1	2	3	
0	77.1	22.4	0.6	0.0	9160
1	14.6	54.7	30.3	0.4	3430
2	0.2	6.0	69.3	24.5	2405
3	0.0	0.1	14.3	85.7	1519
Reviewer A, No.	7563	4069	2976	1906	16 514

^a^ Increasing Mayo endoscopic scores denote increasing mucosal inflammation in the colon, where a score of 0 indicates normal-appearing colonic mucosa and 3 indicates severe inflammatory changes.

### Colonoscopy Video Test Set

Entire colonoscopy videos in 30 sequential patients with UC were recorded; there were 10 Mayo subscores of 0; 7 subscores of 1; 5 subscores of 2; and 8 subscores of 3. Video segmentation resulted in 11 492 images, with a distribution for Mayo subscores as follows: 0, 8148 (70.9%); 1, 1574 (13.7%); 2, 1126 (9.8%); and 3644 (5.6%). Overall, prediction of endoscopic remission vs moderate to severe disease activity using video-based images was excellent, with an AUROC of 0.966 (95% CI, 0.963-0.969) and a positive predictive value of 0.68 (95% CI, 0.67-0.69) and negative predictive value of 0.98 (95% CI, 0.97-0.99) (Figure 3B). Agreement on exact Mayo subscore by the CNN compared with human reference was similar to the still-image model, correctly classifying individual Mayo subscores of 0 in 75.3%; 1 in 67.6%; 2 in 64.3%; and 3 in 67.9% of images with a weighted Cohen κ of 0.75 (95% CI, 0.74-0.76) (eTable 2 in the Supplement).

Finally, in a feasibility pilot, we attempted to predict the summary Mayo subscore for an entire colonoscopy video using the relative proportion of still-image scores. Still-image score proportion threshold rules correctly classified 25 of 30 videos; a summary Mayo subscore of 0 in 10 of 10 cases, Mayo subscore of 1 in 6 of 7 cases (misclassification predicted Mayo subscore of 0), Mayo subscore of 2 in 4 of 5 cases (misclassification predicted Mayo subscore of 1), and Mayo subscore of 3 in 5 of 8 cases (misclassifications as Mayo 2 and 0). The case of a Mayo subscore of 3 being misclassified as a Mayo subscore of 0 resulted from a case of ulcerative proctitis with disease limited to a short distal portion of the large intestine. The mean computational time needed to score an individual colonoscopy video 20 minutes in duration was 18 seconds.

## Discussion

Grading endoscopic severity is used frequently to assess efficacy of new therapies in randomized clinical trials in patients with UC. However, its widespread use in routine clinical practice is limited by the need for experienced human reviewers. We show that deep learning algorithms using CNNs can be trained to grade endoscopic severity with very good discrimination between disease remission and moderate to severe activity. The agreement between deep learning algorithms and adjudicated human scores is actually similar to agreement between independent human reviewers. Finally, although more work is needed before clinical deployment, our feasibility pilot showed that summarizing the overall endoscopic severity within the framework of a full colonoscopy video is also possible.

Reliability of medical image interpretation has long been recognized as a challenge in endoscopy, radiology, and histopathology owing to the subjectivity of individual human reviewers.^22,23^ Specific to UC, Travis and colleagues^9^ found that interobserver agreement of endoscopic severity using colonoscopy videos was good among patients with severe disease (76%), but poor in patients with moderate (37%) and or no disease (27%). As a consequence, blinded central reading in UC is strongly encouraged by regulatory authorities to account for variation in experience, training, consistency, and other biases that can occur with local endoscopist review.^24^ A few studies have directly examined the association of central reading with Mayo endoscopic score accuracy and reproducibility. In a randomized, double-blind, placebo-controlled trial of mesalamine in 281 participants with UC, central reading demonstrated an intraobserver agreement of 0.89 (95% CI, 0.85-0.92) with an interobserver agreement of 0.79 (95% CI, 0.72-0.85).^11^ These overall agreements were similar to the deep learning algorithm using CNN that we present.

Beyond use in clinical trials, automated image analysis has the potential to be readily applied to evaluations that are often hampered by subjectivity across operators. Automated assessments could offer standardized disease activity scoring at scale. Using a commercially available modest graphical processing unit, the CNN presented in this study graded the UC severity in a 20-minute colonoscopy video in 18 seconds; all 30 videos were automatically scored in 9 minutes. The data generated could improve predictive models of clinical outcomes, generate near–real-time postapproval therapeutic efficacy assessments for regulatory agencies, assist payers in value-based assessments of specific treatments, and offer a method for population-level objective disease activity assessments. It could also target specific areas of concern within a long colonoscopy video for additional review by experienced human reviewers.

### Limitations

This work is subject to several limitations. First, deep learning algorithms incorporate any potential bias found in the training set. Although images underwent duplicate independent review with adjudication by a third reviewer (M.D.R.), the subjectivity of the Mayo subscore makes establishing an indisputable “ground truth” challenging even when using experienced human reviewers. In future work, it will be interesting to see whether Mayo subscores generated from deep learning algorithms predict clinical course in individuals better than experienced human reviewers. Second, we used still images obtained from a single health care system, albeit a large referral center for patients with UC. Future studies will need to include a larger set of images collected from a more diverse cohort of patients and health care systems. Third, our colonoscopy videos were captured using high-resolution and 10-bit color depth at 60 frames per second. This allowed for handing of motion blur and interlaced artifacts, but future studies will need to assess the association of varied video capture methods with model accuracy. Finally, although generating summary Mayo endoscopic scores for a colonoscopy video appears to be possible, the sample size is too small to draw a conclusion about the reliability of these methods. The still-image score proportions used for determining overall video score are subject to overfitting. However, all these results support the feasibility of automating scoring of entire colonoscopy videos. Optimization and external validation studies needed to apply CNN approaches in clinical trials or patient care are in progress.

Despite these limitations, we believe our study has important implications. First, we have shown the feasibility of using a CNN to grade the severity of UC with very good to excellent agreement with experienced human reviewers. Expanding access to an objective and reproducible scoring system as accurate as the experts who trained the model is valuable.

Perhaps the greater usefulness of artificial intelligence systems is not in replicating subjective human assessments, but instead redefining the scoring criteria. Existing criterion standards of disease assessment based on human pattern recognition could be improved or surpassed by using artificial intelligence to discern features imperceptible to content experts. A recent study compared deep learning with expert pathologists for detecting lymph node metastasis in patients with breast cancer.^25^ When using immunohistochemistry as the criterion standard in place of expert consensus, deep learning (AUROC, 0.994) outperformed expert pathologists (AUROC, 0.884) in detecting evidence of metastasis on lymph node histology studies.

## Conclusions

We found deep learning algorithms that use CNNs can approximate human grading of endoscopic disease severity in UC. The agreement between the CNN and adjudicated reference scores for individual endoscopic images was similar to the agreement between 2 independent reviewers. Further, colonoscopy videos were able to be analyzed using automated methods and often matched the summary Mayo score provided by human reviewers. At present, the ability of deep learning methods to approximate expert disease assessment has value when considering reproducibility, objectivity, and speed. As we continue to learn best practices and applications in health care, artificial intelligence systems are likely to add to our understanding and treatment of human diseases.

## Notes

Supplement. **eMethods.** Expanded Description of Patient Inclusion and Exclusion Criteria **eTable 1.** Study Cohort Characteristics **eTable 2.** Agreement Between Adjudicated Human Reference Scores and Automated Mayo Subscore Using Images From Colonoscopy Videos
