# Error in Methods, Results, and Table

**DOI:** 10.1001/jamanetworkopen.2019.20585

**Published:** 2020-01-10

In the Original Investigation titled “Performance of a Deep Learning Model vs Human Reviewers in Grading Endoscopic Disease Severity of Patients With Ulcerative Colitis,”^1^ published May 17, 2019, a transcription error resulted in 3 percentages being reported incorrectly in the Results section and Table 1. In the text and Table, Mayo 0 should be 89.0% rather than 89.6%, Mayo 1 should be 52.2% rather than 54.5%, and Mayo 3 should be 73.7% rather than 74.3%. In addition, a description of the quadratic weighting used for the Cohen κ agreement analysis was omitted from the Statistical Analysis subsection of Methods. This article has been corrected.^1^
