# Supplementary material for: Performance of a Deep Learning Model vs Human Reviewers in Grading Endoscopic Disease Severity of Patients With Ulcerative Colitis
Source: JAMA Netw Open. 2019 May 17;2(5):e193963. doi: 10.1001/jamanetworkopen.2019.3963 (PMC6537821; doi:10.1001/jamanetworkopen.2019.3963)
Supplement: Supplement. — eMethods. Expanded Description of Patient Inclusion and Exclusion Criteria eTable 1. Study Cohort Characteristics eTable 2. Agreement Between Adjudicated Human Reference Scores and Automated Mayo Subscore Using Images From Colonoscopy Videos [file jamanetwopen-2-e193963-s001.pdf]

Supplementary Online Content

Stidham RW, Liu W, Bishu S, et al. Performance of a deep learning model vs human reviewers in grading endoscopic disease severity of patients with ulcerative colitis. *JAMA Netw Open*. 2019;2(5):e193963. doi:10.1001/jamanetworkopen.2019.3963

eMethods. Expanded Description of Patient Inclusion and Exclusion Criteria

eTable 1. Study Cohort Characteristics

eTable 2. Agreement Between Adjudicated Human Reference Scores and Automated Mayo Subscore Using Images From Colonoscopy Videos

This supplementary material has been provided by the authors to give readers additional information about their work.

eMethods. Expanded Description of Patient Inclusion and Exclusion Criteria

1. Inclusion Criteria

Study inclusion criteria required two ICD-9 or ICD-10 diagnosis codes for UC on two separate encounters and at least one record of UC medication use.

a. Ulcerative Colitis Diagnosis Codes

**ICD-9 Code**	**Description**
556	Ulcerative (chronic) enterocolitis
556.1	Ulcerative (chronic) ileocolitis
556.2	Ulcerative (chronic) proctitis
556.3	Ulcerative (chronic) proctosigmoiditis
556.5	Left-sided ulcerative (chronic) colitis
556.6	Universal ulcerative (chronic) colitis
556.8	Other ulcerative colitis
556.9	Ulcerative colitis, unspecified
**ICD-10 Code**	**Description**
K51.0x	Ulcerative (chronic) pancolitis
K51.2x	Ulcerative (chronic) proctitis
K51.3x	Ulcerative (chronic) rectosigmoiditis
K51.5x	Left sided colitis
K51.8x	Other ulcerative colitis
K51.9x	Ulcerative colitis, unspecified

b. Ulcerative Colitis Medications

**5-Aminosalicylates**
mesalamine
sulfasalazine
balsalazide
olsalazine
**Corticosteroids**
prednisone
budesonide
**Immunomodulators**
azathioprine
mercaptopurine
methotrexate

cyclosporine
mycophenolate mofetil
tacrolimus
**Biologics**
infliximab
adalimumab
golimumab
vedolizumab

2. Exclusion Criteria

We excluded any patients with ICD-9 or ICD-10 diagnosis codes for Crohn's disease or a CPT code prior to colonoscopy date indicating colectomy, ileoanal pouch anastomosis, colostomy, ileostomy, or other bowel resection.

a. *Crohn's Disease*

**ICD-9 Code**	**Description**
555	Regional enteritis of small intestine
555.1	Regional enteritis of large intestine
555.2	Regional enteritis of small intestine with large intestine
555.9	Regional enteritis of unspecified site
**ICD-10 Code**	**Description**
K50.00x	Crohn's disease of small intestine without complications
K50.011x	Crohn's disease of small intestine with rectal bleeding
K50.012x	Crohn's disease of small intestine with intestinal obstruction
K50.013x	Crohn's disease of small intestine with fistula
K50.014x	Crohn's disease of small intestine with abscess
K50.018x	Crohn's disease of small intestine with other complication
K50.019x	Crohn's disease of small intestine with unspecified complications
K50.10x	Crohn's disease of large intestine without complications
K50.111x	Crohn's disease of large intestine with rectal bleeding
K50.112x	Crohn's disease of large intestine with intestinal obstruction
K50.113x	Crohn's disease of large intestine with fistula
K50.114x	Crohn's disease of large intestine with abscess
K50.118x	Crohn's disease of large intestine with other complication
K50.119x	Crohn's disease of large intestine with unspecified complications
K50.80x	Crohn's disease of both small and large intestine without complications
K50.811x	Crohn's disease of both small and large intestine with rectal bleeding
K50.812x	Crohn's disease of both small and large intestine with intestinal obstruction
K50.813x	Crohn's disease of both small and large intestine with fistula

K50.814x	Crohn's disease of both small and large intestine with abscess
K50.818x	Crohn's disease of both small and large intestine with other complication
K50.819x	Crohn's disease of both small and large intestine with unspecified complications
K50.90x	Crohn's disease, unspecified, without complications
K50.911x	Crohn's disease, unspecified, with rectal bleeding
K50.912x	Crohn's disease, unspecified, with intestinal obstruction
K50.913x	Crohn's disease, unspecified, with fistula
K50.914x	Crohn's disease, unspecified, with abscess
K50.918x	Crohn's disease, unspecified, with other complication
K50.919x	Crohn's disease, unspecified, with unspecified complications

b. *Surgical CPT Procedure Codes*
Procedure codes captured colectomy (total or partial), colostomy, ileostomy, J-pouch or other reservoir procedure. The 2018 NHSN Operative Procedure Code Mapping Tables, maintained by the United States Centers for Disease Control, were used for CPT code reference.

**CPT Code**	**Description**
44139	Mobilization (take-down) of splenic flexure performed in conjunction with partial colectomy
44140	Colectomy, partial; with anastomosis
44141	Colectomy, partial; with skin level cecostomy or colostomy
44143	Colectomy, partial; with end colostomy and closure of distal segment (Hartmann type procedure)
44144	Colectomy, partial; with resection, with colostomy or ileostomy and creation of mucofistula
44145	Colectomy, partial; with coloproctostomy (low pelvic anastomosis)
44146	Colectomy, partial; with coloproctostomy (low pelvic anastomosis), with colostomy
44147	Colectomy, partial; abdominal and transanal approach
44150	Colectomy, total; abdominal, without proctectomy; with ileostomy or ileoproctostomy
44151	Colectomy, total; abdominal, without proctectomy; with continent ileostomy
44155	Colectomy, total; abdominal, with proctectomy; with ileostomy
44156	Colectomy, total; abdominal, with proctectomy; with continent ileostomy
44157	Colectomy, total; abdominal, without proctectomy; with ileoanal anastomosis, includes loop ileostomy
44158	Colectomy, total; abdominal, without proctectomy; with ileoanal anastomosis, creation of ileal reservior
44160	Colectomy, partial; with removal of terminal ileum with ileocolostomy $1,295
44320	Colostomy or skin level cecostomy
44322	Colostomy or skin level cecostomy; with multiple biopsies
45110	Proctectomy; complete, combined abdominoperineal, with colostomy
45111	Proctectomy; partial resection of rectum, transabdominal approach
45112	Proctectomy, combined abdominoperineal, pull-through procedure

45113	Proctectomy, partial, with rectal mucosectomy, ileoanal anastomosis, creation of ileal reservoir
45114	Proctectomy, partial, with anastomosis; abdominal and transsacral approach
45119	Proctectomy, combined abdominoperineal pull-through procedure (eg, colo-anal anastomosis), with creation of colonic reservoir (eg, J-pouch), with diverting enterostomy when performed
45120	Proctectomy, complete (for congenital megacolon), abdominal and perineal approach; with pull-through
45121	Proctectomy, complete (for congenital megacolon), abdominal and perineal approach
45123	Proctectomy, partial, without anastomosis, perineal approach
45399	Unlisted procedure, colon
44320	Under Enterostomy-External Fistulization of Intestines Procedures
44204	Laparoscopy, surgical; colectomy, partial, with anastomosis
44205	Laparoscopy, surgical; colectomy, partial, with removal of terminal ileum with ileocolostomy
44206	Laparoscopy, surgical; colectomy, partial, with end colostomy and closure of distal segment (Hartmann type procedure)
44207	Laparoscopy, surgical; colectomy, partial, with anastomosis, with coloproctostomy (low pelvic anastomosis)
44208	Laparoscopy, surgical; colectomy, partial, with anastomosis, with coloproctostomy (low pelvic anastomosis) with colostomy
44210	Laparoscopy, surgical; colectomy, total, abdominal, without proctectomy, with ileostomy or ileoproctostomy
44211	Laparoscopy, surgical; colectomy, total, abdominal, with proctectomy, with ileoanal anastomosis, creation of ileal reservoir (S or J), with loop ileostomy, includes rectal mucosectomy, when performed
44212	Laparoscopy, surgical; colectomy, total, abdominal, with proctectomy, with ileostomy
45136	Excision of ileoanal reservoir with ileostomy
44314	Revision of ileostomy; complicated (reconstruction in-depth) (separate procedure)
44316	Continent ileostomy (Kock procedure) (separate procedure)
44310	Ileostomy or jejunostomy, non-tube
44312	Revision of ileostomy; simple (release of superficial scar) (separate procedure)

eTable 1. Study Cohort Characteristics

Age, median years (IQR)	41.3	26.1, 61.8
Gender, male	1404	45.6%
**Race**		
White	2720	88.3%
African American	163	5.3%
Asian	102	3.3%
Pacific Islander	33	1.1%
Native American	6	0.2%
Unreported	59	1.9%
**Ethnicity**		
Non-Hispanic	2784	90.3%
Hispanic	168	5.5%
Unreported	130	4.2%
Smoking History	942	30.6%
**Medication Exposure***		
Mesalamine	2275	73.8%
Thiopurine	907	29.4%
Methotrexate	105	3.4%
Infliximab	509	16.5%
Adalimumab	434	14.1%
Vedolizumab	175	5.7%

*****Percentages do not add up to 100% due to overlap in medication use

eTable 2. Agreement Between Adjudicated Human Reference Scores and Automated Mayo Subscore Using Images From Colonoscopy Videos

HUMAN Mayo Score	PREDICTED Mayo Score, %				
	0	1	2	3	Human Total *(n)*
0	**75.3**	20.6	3.1	1.0	8143
1	12.7	**67.6**	18.9	0.7	1578
2	1.9	15.9	**64.3**	18.0	1123
3	2.5	2.0	27.6	**67.9**	648
Predicted Total *(n)*	6370	2934	1451	737	11,492

Increasing Mayo endoscopic scores denote increasing mucosal inflammation in the colon, where a score of 0 indicates normal appearing colonic mucosa and 3 indicates severe inflammatory changes.
